# Neuroprotective Agents With Reperfusion Therapies in Ischemic Stroke: Evidence From Recent Randomized Trials

**DOI:** 10.7759/cureus.88443

**Published:** 2025-07-21

**Authors:** Moses John Wesley, Hamesh Gundala Raja, Muhammad Salman Shahid, Almas Akbar, Deepapriya Jeyakumar, Elakkiya Veluchamy, Manoj Sivakumar, Fizza Tahir, Diego Jiménez Royg, Ramesh Pant, Abdur Rehman

**Affiliations:** 1 Internal Medicine, Annai Hospital, Thuraiyur, IND; 2 Medicine and Surgery, K.A.P. Viswanatham Government Medical College, Tiruchirappalli, IND; 3 Internal Medicine, King Edward Medical University, Lahore, PAK; 4 Internal Medicine, Rawalpindi Medical University, Rawalpindi, PAK; 5 Internal Medicine, Government Sivagangai Medical College and Hospital, Sivaganga, IND; 6 General Practice, Sree Mookambika Institute of Medical Sciences, Kanyakumari, IND; 7 Internal Medicine, K.A.P. Viswanatham Government Medical College, Tiruchirappalli, IND; 8 Internal Medicine, National University of Asunción, San Lorenzo, PRY; 9 Internal Medicine, Maya Metro Hospital Pvt. Ltd., Dhangadhi, NPL; 10 Medicine and Surgery, King Edward Medical University, Lahore, PAK

**Keywords:** acute ischemic stroke, adjunctive therapy, edaravone, infarct volume, modified rankin scale, neuroprotection, normobaric hyperoxia, randomized controlled trials, thrombectomy, thrombolysis

## Abstract

This systematic review evaluates the effectiveness and safety of adjunctive neuroprotective therapies administered in combination with reperfusion treatments, either intravenous (IV) thrombolysis, mechanical thrombectomy, or both, in adult patients with acute ischemic stroke (AIS). Following Preferred Reporting Items for Systematic Reviews and Meta-Analyses (PRISMA) guidelines, a comprehensive search of four major databases was conducted with filters applied to include English-language randomized controlled trials (RCTs) published within the last five years. From an initial 622 records, 10 eligible RCTs were identified and analyzed. Interventions included nerinetide, edaravone (and its derivatives), nelonemdaz, cerebrolysin, minocycline, glyceryl trinitrate, and normobaric hyperoxia. Functional outcomes were primarily measured using the modified Rankin Scale (mRS), infarct volume, and safety endpoints such as symptomatic intracranial hemorrhage (sICH). Among the findings, normobaric hyperoxia showed the most consistent benefit in reducing infarct size and improving functional outcomes, while agents such as nerinetide and edaravone demonstrated mixed results. Quality assessment using the Cochrane Risk of Bias (RoB) 2.0 tool revealed low to moderate risk of bias across most studies. Although the evidence remains heterogeneous, this review highlights the potential role of neuroprotective agents as adjuncts to reperfusion therapy and identifies promising directions for future clinical trials.

## Introduction and background

Acute ischemic stroke (AIS) remains a major cause of long-term disability and mortality worldwide, driven by the sudden occlusion of cerebral arteries resulting in irreversible neuronal injury [1]. The cornerstone of current management involves rapid restoration of cerebral perfusion using intravenous (IV) thrombolysis with tissue plasminogen activator (tPA) or endovascular thrombectomy (EVT) in eligible patients. While these reperfusion strategies have transformed acute stroke care by improving functional outcomes, they are not universally effective [2]. Many patients fail to achieve substantial recovery due to downstream consequences such as reperfusion injury, inflammation, excitotoxicity, and blood-brain barrier disruption. These pathophysiological processes highlight an urgent need to explore adjunctive interventions that target neuronal survival in the vulnerable peri-infarct zone.

Neuroprotection has long been proposed as a strategy to enhance stroke outcomes by mitigating secondary neuronal injury and extending the therapeutic time window. Over the past decade, particularly in the last five years, advances in pharmacological development, translational neuroscience, and neuroimaging technologies have facilitated the identification and clinical testing of several promising neuroprotective agents. These include glutamate antagonists, free radical scavengers, anti-inflammatory molecules, and agents that enhance cerebral oxygen delivery [3,4]. Among them, compounds such as nerinetide, edaravone, minocycline, cerebrolysin, and nelonemdaz have garnered interest due to their favorable pharmacokinetics and mechanistic rationale [5]. In parallel, non-pharmacological approaches such as normobaric hyperoxia and induced hypothermia have also shown potential as adjunctive neuroprotective strategies when combined with reperfusion therapies [6]. Several randomized clinical trials published within the past five years have evaluated these agents as adjuncts to intravenous thrombolysis, mechanical thrombectomy, or both. However, the results remain variable, and the overall clinical utility of these strategies continues to be debated. To address this gap, we conducted a systematic review of randomized controlled trials (RCTs) identified through comprehensive searches of PubMed, Embase, Scopus, and Web of Science published in the last five years. This review aims to evaluate and compare the effectiveness and safety of adjunctive neuroprotective therapies administered alongside reperfusion treatments in adults with acute ischemic stroke, synthesizing recent evidence to inform clinical decision-making and guide future research.

## Review

Materials and methods

Eligibility Framework and Search Strategy

This systematic review was developed using the PICO framework [7], which provided a structured approach to formulate the research question and define study eligibility criteria. Specifically, the population (P) was defined as adult patients diagnosed with acute ischemic stroke who were eligible to receive reperfusion therapy. The intervention (I) included any pharmacological or non-pharmacological neuroprotective strategy administered in conjunction with reperfusion treatments, such as intravenous thrombolysis, mechanical thrombectomy, or both. The comparison (C) consisted of standard reperfusion therapy alone or in combination with a placebo, serving as the control group. The outcomes (O) of interest included clinically relevant endpoints such as functional recovery (typically assessed by the modified Rankin Scale (mRS)), infarct volume (measured via neuroimaging), all-cause mortality, and adverse events including symptomatic intracranial hemorrhage. These PICO elements were systematically applied during the study selection process to include only randomized controlled trials that matched these predefined criteria and focused on adjunctive neuroprotection in the setting of reperfusion therapy.

The search strategy for this systematic review was conducted in accordance with Preferred Reporting Items for Systematic Reviews and Meta-Analyses (PRISMA) guidelines [8] to ensure a comprehensive, transparent, and replicable process. Major databases, including PubMed, Scopus, Embase, and Web of Science, were systematically searched to identify relevant studies evaluating adjunctive neuroprotective therapies administered alongside reperfusion treatments in acute ischemic stroke. To maintain clinical relevance and currency, filters were applied to include only studies published in the last five years, written in English, and classified as clinical trials. The screening process involved title and abstract review, followed by full-text assessment, ensuring that only randomized controlled trials evaluating neuroprotective interventions in combination with thrombolysis or mechanical thrombectomy were included. This rigorous strategy ensured the inclusion of high-quality and recent evidence aligned with the objectives of the review.

Eligibility Criteria

Studies were included in this systematic review based on predefined eligibility criteria aligned with the research objective. Only randomized controlled trials (RCTs) evaluating adjunctive neuroprotective therapies administered in combination with reperfusion treatment, either thrombolysis, thrombectomy, or both, in adult patients with acute ischemic stroke were considered. Eligible studies were required to report at least one clinical or imaging outcome, such as infarct volume, functional recovery, or mortality. The time frame for inclusion was from January 1, 2019, to March 15, 2024, ensuring the synthesis of contemporary evidence. Only articles published in English were included. To ensure appropriate classification, studies were identified and filtered as clinical trials using predefined database filters (e.g., “clinical trial” or “randomized controlled trial” in PubMed, Embase, Scopus, and Web of Science) and then manually verified during full-text screening based on study design, methodology, and outcome reporting. Studies involving pediatric populations, non-randomized designs, animal models, or lacking a comparator arm were excluded from the final synthesis.

Data Extraction

Data extraction was carried out independently by multiple reviewers using a pre-piloted, standardized data extraction form to ensure consistency and minimize bias across studies. This structured tool was designed in alignment with the study objectives and tested on a sample of eligible trials before full implementation. For each included study, key characteristics were systematically collected, including author and year, study design, sample size, intervention details (neuroprotective agent, dosage, and duration), reperfusion method (thrombolysis and/or thrombectomy), comparator used, primary and secondary outcomes, and major findings. Additional elements such as imaging endpoints, statistical significance, and timing of intervention administration were also recorded. Discrepancies in data extraction were resolved through discussion among reviewers, and where necessary, full-text re-evaluation was performed to clarify ambiguous or missing data.

Data Analysis and Synthesis

Given the clinical and methodological heterogeneity across studies, including variations in intervention type, dosage, timing, and outcome measurement, a narrative synthesis approach was adopted. The results were grouped thematically based on the neuroprotective agent used and associated outcomes, such as infarct volume, functional recovery, and safety indicators. Comparative trends and patterns were described qualitatively to highlight the consistency or variability of effects. Although a formal meta-analysis was not feasible, structured tabulation and critical appraisal were employed to integrate findings. The critical appraisal, conducted using the Cochrane Risk of Bias (RoB) 2.0 tool, informed the synthesis by identifying potential sources of bias, which were considered when interpreting outcome consistency and clinical relevance. Furthermore, the strength and certainty of the evidence for each outcome were qualitatively assessed based on methodological rigor, sample size, outcome validity, and consistency across trials, allowing for a more nuanced interpretation of the overall findings.

Results

Study Selection Process

**Figure 1:**
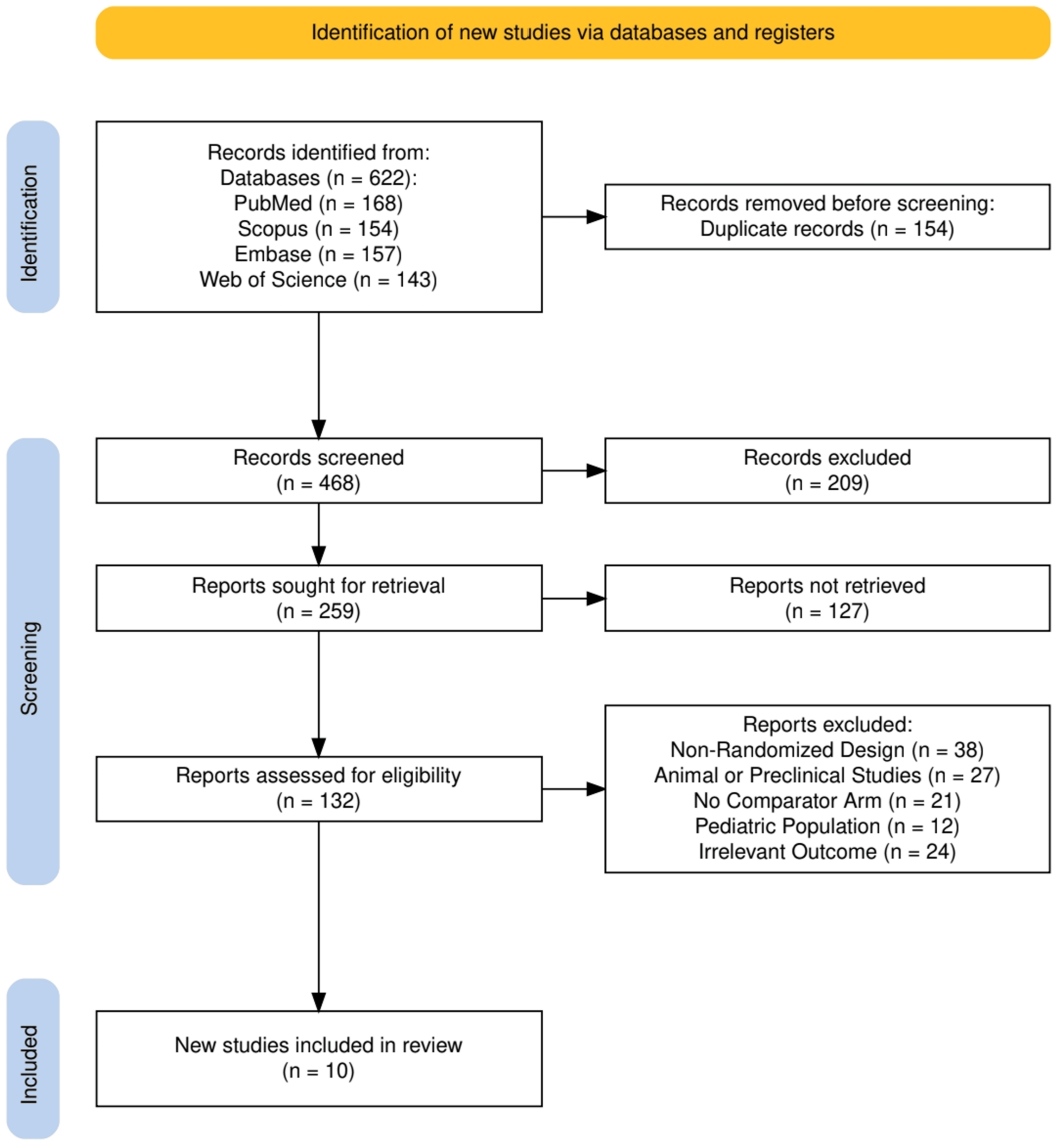
PRISMA flow diagram illustrating the process of the study selection PRISMA: Preferred Reporting Items for Systematic Reviews and Meta-Analyses

The study selection process is illustrated in Figure 1, which outlines the PRISMA flow diagram used to document the identification, screening, and inclusion of studies for this systematic review. A total of 622 records were initially identified through database searches (PubMed: 168, Scopus: 154, Embase: 157, and Web of Science: 143). After removing 154 duplicate entries, 468 records underwent title and abstract screening, resulting in 209 exclusions based on relevance. The remaining 259 reports were sought for full-text retrieval, of which 127 could not be retrieved, leaving 132 reports for eligibility assessment. Of these, 122 were excluded for reasons including non-randomized design (38), preclinical/animal studies (27), lack of a comparator arm (21), pediatric population (12), and irrelevant outcomes (24). Ultimately, 10 randomized controlled trials met all inclusion criteria and were included in the final synthesis. This structured and transparent approach, guided by PRISMA methodology, ensured the inclusion of high-quality evidence relevant to the review objectives.

Characteristics of the Selected Studies

The characteristics of the selected studies are summarized in Table 1, reflecting a diverse range of randomized controlled trials evaluating neuroprotective agents administered alongside reperfusion therapies in acute ischemic stroke. The sample sizes of the included studies varied widely, from smaller pilot trials with fewer than 50 participants to large multicenter investigations enrolling over 800 patients. Most studies employed a placebo or standard care comparator and infarct volume as primary outcome measures. Interventions included pharmacological agents such as nerinetide, edaravone (and its dexborneol formulation), cerebrolysin, minocycline, and nelonemdaz, as well as non-pharmacological strategies such as high-flow normobaric oxygen and intra-arterial glyceryl trinitrate. Reperfusion strategies consisted primarily of mechanical thrombectomy, with or without thrombolysis, or intravenous thrombolysis alone. While some trials reported significant reductions in infarct volume or trends toward improved functional outcomes, others demonstrated no statistically significant benefit, highlighting variability in effectiveness across agents and study designs. One study remains ongoing with results pending. Overall, the table provides a comprehensive view of the methodologies, interventions, and key findings that form the basis of this systematic analysis.

**Table 1:** Summary of randomized controlled trials evaluating neuroprotective strategies combined with reperfusion therapies in acute ischemic stroke AIS: acute ischemic stroke, EVT: endovascular thrombectomy, IV: intravenous, mRS: modified Rankin Scale, DWI: diffusion-weighted imaging, MRI: magnetic resonance imaging, sICH: symptomatic intracranial hemorrhage, NIHSS: National Institutes of Health Stroke Scale, NBO: normobaric oxygen, AE: adverse events, FiO₂: fraction of inspired oxygen, OR: odds ratio, CI: confidence interval, BID: twice daily, RCT: randomized controlled trial, sHT: symptomatic hemorrhagic transformation

Study (author, year)	Study design	Sample size	Intervention (neuroprotective agent)	Reperfusion method	Comparator	Primary outcome(s)	Key findings
Hill et al. (2025) (ESCAPE-NEXT) [9]	Multicenter RCT, double-blind	850	Nerinetide (2.6 mg/kg IV, single dose)	EVT (no prior thrombolysis)	Placebo	mRS 0-2 at 90 days	No significant difference between groups (OR: 0.97, 95% CI: 0.72-1.30; p=0.82)
Fladt et al. (2024) (REPERFUSE-NA1) [10]	Prospective RCT sub-study, MRI-based analysis	71	Nerinetide (IV)	EVT (some with alteplase)	Placebo	Infarct growth on DWI MRI	No significant reduction overall; reduced growth in white matter and basal ganglia without alteplase (p=0.03 and p=0.02)
Chen et al. (2025) [11]	Phase II RCT, double-blind	200	Edaravone dexborneol (37.5 mg IV BID ×12 days)	Thrombectomy (successful reperfusion)	Placebo	mRS 0-2 at 90 days	58.7% versus 52.1% mRS 0-2; not statistically significant (OR: 1.37; p=0.29)
Hong et al. (2022) (SONIC) [12]	Phase II RCT, multicenter	208	Nelonemdaz (low-dose: 2,750 mg, high-dose: 5,250 mg)	EVT within 8 hours	Placebo	mRS 0-2 at 12 weeks	No significant difference; favorable trend (OR: 1.55-1.61); no serious AEs
Li et al. (2022) [13]	Pilot RCT, assessor-blinded	86	Normobaric hyperoxia (100% O₂, 10 L/minute ×4 hours pre-EVT)	EVT	Room air (EVT only)	Infarct volume (MRI 24-48 hours)	Infarct volume significantly reduced (20.1 versus 37.7 mL; p<0.01); improved mRS (p=0.038)
Cheng et al. (2023) (AGAIN) [14]	Pilot RCT, single-center	40	Glyceryl trinitrate (800 μg intra-arterial post-EVT)	Post-thrombectomy	Normal saline	sICH, mRS, infarct volume	No increase in sICH; trends toward better mRS and smaller infarct volume
Zhang et al. (2024) (MIST-A) [15]	Prospective RCT, open-label, blinded-endpoint	180 (planned)	Minocycline + standard care	Intravenous thrombectomy (anterior circulation)	Standard care	Change in infarct volume (baseline to day 5)	Trial ongoing; results pending
Khasanova et al. (2023) (CEREHETIS) [16]	Open-label RCT, multicenter pilot	341	Cerebrolysin + alteplase	Thrombolysis	Alteplase alone	Symptomatic hemorrhagic transformation	Reduced sHT (OR: 0.248; p=0.019); better NIHSS at day 14; no mRS difference at day 90
Li et al. (2020) [17]	RCT, single-center	118	Edaravone + alteplase	Thrombolysis	Alteplase alone	Neurological, T-cells, hemodynamics	Improved neurological scores and hemodynamics (p<0.05)
Cheng et al. (2021) [18]	Prospective RCT	91	High-Flow NBO (FiO₂ 50%, 15 L/minute ×6 hours)	Thrombectomy (anterior circulation)	Low-flow oxygen (3 L/minute)	mRS at 90 days, infarct volume, mortality	Improved mRS (OR: 2.2); lower infarct volume and mortality; no excess complications

Quality Assessment

The quality assessment of the included studies, summarized in Table 2, was conducted using the Cochrane Risk of Bias (RoB) 2.0 tool [19] and revealed a mixed level of methodological rigor across trials. Several high-quality studies, such as those evaluating nerinetide, edaravone dexborneol, nelonemdaz, and high-flow normobaric oxygen, demonstrated a low risk of bias across all assessed domains, indicating strong internal validity. However, a few studies, including the MIST-A and CEREHETIS trials, were judged as having “some concerns,” which in the context of the Cochrane RoB 2.0 tool refers to uncertainty in at least one domain that raises the possibility of bias, but not enough to be classified as high risk. These concerns typically arose from issues such as deviations from intended interventions, incomplete outcome data, or lack of blinding. One study involving edaravone combined with alteplase was assessed as having a high overall risk of bias, primarily due to concerns in multiple domains, including selective outcome reporting and lack of concealment. Despite this variability, the overall quality of evidence was adequate to support meaningful narrative synthesis and interpretation. Nonetheless, these findings highlight the need for more uniformly rigorous and blinded trial designs in future research.

**Table 2:** Summary of study-level risk of bias judgments based on the Cochrane RoB 2.0 tool RoB: Risk of Bias, RCT: randomized controlled trial, EVT: endovascular thrombectomy, mRS: modified Rankin Scale

Study (author, year)	Tool used	Randomization process	Deviations from intended interventions	Missing outcome data	Measurement of the outcome	Selection of the reported result	Overall risk of bias
Hill et al. (2025) (ESCAPE-NEXT) [9]	RoB 2.0	Low	Low	Low	Low	Low	Low
Fladt et al. (2024) (REPERFUSE-NA1) [10]	RoB 2.0	Low	Low	Some concerns	Low	Some concerns	Some concerns
Chen et al. (2025) [11]	RoB 2.0	Low	Low	Low	Low	Low	Low
Hong et al. (2022) (SONIC) [12]	RoB 2.0	Low	Low	Low	Low	Low	Low
Li et al. (2022) [13]	RoB 2.0	Low	Some concerns	Low	Low	Low	Some concerns
Cheng et al. (2023) (AGAIN) [14]	RoB 2.0	Some concerns	Low	Low	Low	Low	Some concerns
Zhang et al. (2024) (MIST-A) [15]	RoB 2.0	Low	Some concerns	Some concerns	Some concerns	Some concerns	Some concerns
Khasanova et al. (2023) (CEREHETIS) [16]	RoB 2.0	Some concerns	High	Low	Low	Some concerns	High
Li et al. (2020) [17]	RoB 2.0	Some concerns	High	Some concerns	Some concerns	High	High
Cheng et al. (2021) [18]	RoB 2.0	Low	Low	Low	Low	Low	Low

Discussion

This systematic review evaluated the efficacy of adjunctive neuroprotective therapies administered alongside reperfusion treatments, either thrombolysis or mechanical thrombectomy, in patients with acute ischemic stroke. Among the 10 included randomized controlled trials [9-18], the most consistent benefits were observed with normobaric hyperoxia, which significantly reduced infarct volume and improved functional outcomes as measured by the modified Rankin Scale (mRS) [20] at 90 days. High-flow oxygen therapy post-thrombectomy, in particular, was associated with both decreased mortality and favorable neurological recovery. Edaravone-based interventions, including edaravone dexborneol, demonstrated modest improvements in early neurological function, most commonly assessed using the National Institutes of Health Stroke Scale (NIHSS). However, the consistency of these benefits varied: while some studies reported significant improvements in early NIHSS scores and imaging markers such as reduced infarct volume, these did not consistently translate into statistically significant improvements in long-term functional outcomes (e.g., mRS 0-2 at 90 days). Nerinetide showed no overall benefit in large trials, but subgroup analyses indicated a potential reduction in infarct volume in patients not treated with alteplase. Agents such as nelonemdaz and cerebrolysin exhibited favorable trends and strong mechanistic plausibility, although definitive clinical benefits remain unconfirmed. Across trials, infarct volume reduction and mRS-based functional recovery were the outcomes most influenced by adjunctive neuroprotective therapy, with acceptable safety profiles consistently reported.

Compared to previous systematic reviews and meta-analyses, this study presents a more updated and targeted synthesis of adjunctive neuroprotective strategies administered alongside reperfusion therapies in acute ischemic stroke [21]. Prior literature often yielded inconclusive results due to the pooling of heterogeneous interventions and reliance on outdated data, which frequently involved older reperfusion protocols, limited use of advanced neuroimaging, and inclusion of trials conducted before widespread adoption of endovascular thrombectomy. Our findings, however, underscore that normobaric hyperoxia consistently demonstrated significant reductions in infarct volume and modest functional improvements [22], aligning with earlier pilot studies but now supported by more recent, higher-quality evidence. In contrast, nerinetide and edaravone trials yielded mixed results, echoing inconsistencies seen in earlier evaluations. For nerinetide, previous studies reported no significant benefit when co-administered with alteplase, potentially due to drug-cleaving interactions with plasmin, while showing some infarct volume reduction in patients who did not receive thrombolysis. Edaravone, despite its antioxidant properties and early promise in Asian populations, showed variability in long-term functional outcomes, with some trials reporting improvements in early neurological scores but no consistent benefit in 90-day mRS results [23]. Discrepancies in outcomes such as modified Rankin Scale scores and infarct volumes may also be attributed to variations in dosage, timing of administration relative to recanalization, inclusion criteria (e.g., large vessel occlusion versus general AIS), and methodological factors such as sample size or blinding protocols [24]. These variations emphasize the need for stratified, standardized, and imaging-guided research designs in future trials.

The clinical applicability of adjunctive neuroprotective therapies in acute ischemic stroke remains cautiously optimistic. Normobaric oxygen, being inexpensive, non-invasive, and widely available, stands out as a highly translatable option, particularly in low-resource settings or as an immediate prehospital intervention. Edaravone-based compounds, while requiring intravenous administration over several days, may hold value in hospital-based stroke units as supportive therapies, especially where early reperfusion has been achieved [25]. Despite the lack of statistically significant benefit in many trials, the consistently favorable safety profiles of agents such as nerinetide, cerebrolysin, and minocycline support their continued investigation. These therapies, particularly when guided by imaging or tailored to specific patient subgroups, may find their place in future stroke management algorithms, potentially as adjuncts for patients at high risk of reperfusion injury or with early imaging evidence of salvageable tissue. However, this optimism must be tempered by the mixed efficacy results across trials and the methodological heterogeneity observed, including variability in trial design, sample size, blinding, and outcome assessment. These limitations underscore the need for more robust, standardized clinical trials before widespread clinical adoption.

The observed therapeutic effects of the included neuroprotective agents can be traced to their mechanistic actions on the ischemic cascade. Edaravone and its derivatives function as potent free radical scavengers, reducing oxidative stress and stabilizing the blood-brain barrier, an effect that may account for the reduced infarct volumes and improved neurological indices seen in some trials [26]. Similarly, normobaric hyperoxia enhances oxygen delivery to the ischemic penumbra, preserving neuronal viability until reperfusion is achieved, which likely explains its consistent association with reduced infarct size and improved outcomes. Nelonemdaz, a dual-action N-methyl-D-aspartate (NMDA) receptor antagonist and antioxidant, theoretically offers both excitotoxic and oxidative injury mitigation, although its clinical effects were modest [27]. Glyceryl trinitrate may confer benefit by modulating cerebral blood flow and nitric oxide levels post-recanalization [28]. These insights underscore the biologic plausibility of neuroprotection in acute stroke and support further refinement of these agents within pathophysiologically targeted strategies.

The quality assessment of the included studies, conducted using the Cochrane RoB 2.0 tool [19], revealed a moderate level of overall confidence in the pooled evidence. While several trials, such as ESCAPE-NEXT [9] and SONIC [12], and the high-flow normobaric oxygen studies demonstrated low risk of bias across most domains, others raised methodological concerns. Specifically, trials such as CEREHETIS [16] and the edaravone-alteplase study [11] exhibited higher risk due to open-label designs, potential deviations from intended interventions, and selective reporting. Additionally, several studies were single-center or pilot in nature with relatively small sample sizes, which may limit the robustness and generalizability of their findings. These limitations necessitate caution when interpreting individual trial outcomes and underscore the importance of methodological rigor in future research.

This review offers several strengths that enhance its reliability and relevance. The methodology was firmly grounded in PRISMA guidelines, ensuring a transparent and reproducible approach to study selection and data synthesis. A comprehensive and targeted search strategy across major databases allowed the inclusion of recent, high-quality randomized controlled trials, many of which were published within the past five years. The use of a structured risk of bias tool added further validity to the evaluation of evidence. However, this review is not without limitations. The included studies varied considerably in terms of neuroprotective agent, dosage regimen, outcome measures, and imaging protocols, leading to significant heterogeneity. Moreover, the absence of a formal meta-analysis limits the ability to provide quantitative effect estimates. The inclusion of early-phase and single-center trials also affects the external validity of the findings, and the inability to pool results due to inconsistent endpoints further challenges the derivation of definitive conclusions.

Looking forward, future research should focus on large, multicenter, double-blind phase III randomized controlled trials to establish the efficacy and safety of adjunctive neuroprotective agents with greater statistical power and external validity. Combination approaches that simultaneously target oxidative stress, excitotoxicity, and inflammation, such as pairing antioxidants with anti-inflammatory agents, may offer synergistic benefits and warrant exploration. Additionally, stratified trial designs based on stroke subtypes, perfusion imaging profiles, and time-to-treatment windows could help identify patient subgroups most likely to benefit from specific neuroprotective strategies. Incorporating advanced biomarkers and functional imaging may further aid in tailoring therapies and elucidating mechanistic pathways, ultimately advancing the clinical translation of neuroprotection in acute stroke care.

## Conclusions

This systematic review highlights the evolving landscape of adjunctive neuroprotective therapies in acute ischemic stroke management, revealing both the promise and the limitations of current clinical evidence. While no single agent demonstrated definitive superiority across all outcomes, interventions such as high-flow normobaric oxygen and edaravone-based therapies showed meaningful trends toward reduced infarct volumes and improved functional recovery when used alongside thrombolysis or thrombectomy. The consistently favorable safety profiles and mechanistic rationale behind these therapies reinforce their potential role in complementing reperfusion strategies. This review is significant as it consolidates contemporary clinical trials, critically appraises their quality, and provides a structured synthesis that may inform both future research directions and early-stage clinical implementation. By identifying gaps in evidence and proposing refined trial frameworks, this study serves as a foundational reference for advancing the integration of neuroprotection into standard stroke care.

## Appendices

Copyright and permissions clarification

This systematic review utilized the following established tools and frameworks, all of which are permissible for academic use under fair use policies or open-access guidelines.

PICO Framework

The PICO (Population, Intervention, Comparison, and Outcome) framework was used to structure the research question and guide eligibility criteria. This framework is a well-established educational tool in evidence-based medicine and does not require permission for academic or research use. It has been properly cited as follows: Richardson WS, Wilson MC, Nishikawa J, Hayward RS: The well-built clinical question: a key to evidence-based decisions. ACP J Club. 1995, 123:A12-3 [7].

Modified Rankin Scale (mRS)

The modified Rankin Scale was employed solely for reporting clinical outcomes (e.g., mRS 0-2 at 90 days). The scale was not reproduced, adapted, or modified in any form. The standard citation for interobserver reliability was provided as follows: van Swieten JC, Koudstaal PJ, Visser MC, Schouten HJ, van Gijn J: Interobserver agreement for the assessment of handicap in stroke patients. Stroke. 1988, 19:604-7. 10.1161/01.str.19.5.604 [20].

Cochrane Risk of Bias 2.0 (RoB 2.0) Tool

The Cochrane RoB 2.0 tool was used for assessing the methodological quality of included randomized controlled trials. No proprietary diagrams, checklists, or modifications of the tool were included. The tool was used in accordance with Cochrane Collaboration’s guidelines for academic use, and cited as follows: Sterne JA, Savović J, Page MJ, et al.: RoB 2: a revised tool for assessing risk of bias in randomised trials. BMJ. 2019, 366:l4898. 10.1136/bmj.l4898 [19].

No part of the manuscript reproduces copyrighted scales, structured interviews, or tool visuals. All references are appropriately cited, and the use of these instruments complies with fair academic usage.
